# Energy-Efficient Adaptive Bidirectional Transmission Strategy in Simultaneous Wireless Information and Power Transfer (SWIPT)-Enabled Cognitive Relay Network

**DOI:** 10.3390/s24196478

**Published:** 2024-10-08

**Authors:** Caixia Cai, Jiayao Zhang, Fuli Zhong, Han Hai

**Affiliations:** 1 Department of Electrical Engineering, Shanghai Maritime University, Shanghai 201306, China; jyzhangyyy@163.com; 2 School of Systems Science and Engineering, Sun Yat-sen University, Guangzhou 510275, China; zhongfulicn@163.com; 3 College of Information Sciences and Technology, Donghua University, Shanghai 201620, China; hhai@dhu.edu.cn

**Keywords:** simultaneous wireless information and power transfer, adaptive transmission, optimal power allocation, energy efficiency, spectrum efficiency

## Abstract

Introducing collaborative relay and simultaneous wireless information and power transfer (SWIPT) techniques into a cognitive wireless network, named the SWIPT-enabled cognitive relay network (CRN), is considered a promising approach to deal with insufficiency and the low utilization of spectrum resources, as well as the node’s energy-constrained issues in wireless networks. In this paper, to improve the network spectrum efficiency (SE) and energy efficiency (EE) of the SWIPT-enabled CRN, we design an energy-efficient adaptive bidirectional transmission strategy. To be specific, we first select an energy-constrained best relay node with the consideration of signal-to-noise ratio and global channel gain to achieve a better bidirectional relay transmission (BRT). At the same time, we let the energy-constrained best relay node transmit a signal with the SWIPT technique, which can solve the node’s energy-constrained issue and improve the network EE. Then, with the selected energy-constrained best relay node, we design a total transmit power threshold (TTPT) determining algorithm to find the TTPT, which lets the total transmission rate of the BRT be equal to the bidirectional direct transmission (BDT). Based on this TTPT, we further design an adaptive bidirectional transmission strategy and let the network achieve adaptive transmission between the BRT and BDT to obtain a higher network SE. Furthermore, to further achieve the energy-efficient transmission of the adaptive bidirectional transmission strategy, we optimize the nodes’ power under the requirement of primary users’ interference threshold and obtain the analytical expressions of the optimal power. Simulation results show that the transmission rate, the outage probability, and the EE of the designed energy-efficient adaptive bidirectional transmission strategy in the SWIPT-enabled CRN are, respectively, 3.01, 0.07, and 3.10 times that of the non-collaborative transmission, which show the effectiveness of our designed transmission strategy.

## 1. Introduction

The collaborative relay technique is an effective technique to achieve higher spectrum efficiency (SE) due to its ability to save the node’s power consumption and realize the spatial diversity [1,2]. The authors in [3,4] propose a unidirectional decode-and-forward relay transmission scheme because it can increase the transmission rate and reduce the bit error rate. However, unidirectional relay transmission uses two time slots to complete one direction signal transmission, which reduces the time slot utilization and causes the SE loss [5]. For this problem, the authors in [6,7] propose a bidirectional relay transmission scheme that utilizes two time slots for bidirectional signals exchange. However, refs. [6,7] are based on single relay transmission. Under single relay transmission, once the relay node fails, the entire relay link will be subject to outage. Subsequently, the authors in [8,9] propose a bidirectional multi-relay (BMR) transmission strategy, which improves the network SE while increasing the time slot utilization.

In the BMR transmission, the channel quality on either side of each relay node differs, which may result in communication outages or insufficient channel capacity. Therefore, it is crucial to select the best relay node(s) to achieve reliable transmission. In [10], the authors propose a relay selection algorithm based on the signal-to-noise ratio (SNR) threshold. However, since this algorithm only relies on SNR for relay selection, it may allow interfering nodes to act as relays, thus affecting the quality of signal transmission. In [11], the authors use an opportunity relay selection algorithm to transmit signals. Although this algorithm provides better performance in some cases, it increases the complexity and management cost of the network. And it is also difficult to cope with dynamic network environments. For these problems, the authors in [12] propose an optimal relay selection algorithm based on SNR and channel gain, thus improving the transmission rate. However, ref. [12] only considers the single channel gain to be better, while neglecting the case that the global channel gain should be better. Meanwhile, refs. [10,11,12] only use the relay link to transmit signals. It means that they neglect the potential of direct link (DL) being better. The DL means the link directly between the transmit and receive nodes [13].

Besides the collaborative relay technique, the cognitive wireless network (CWN) allows secondary users (SUs) to share spectrum resources with primary users (PUs) without affecting the normal communication of the PUs, which can also improve spectrum utilization [14,15]. Considering the advantages of CWN and collaborative relay technique, combining CWN with the collaborative relay technique to construct a cognitive relay network (CRN) has continued to receive extensive attention from researchers. In [16], the authors study an adaptive cooperative transmission strategy in cognitive bidirectional relay network to improve the performance of the SUs. At the same time, the optimal relay selection approach for this bidirectional relay network to minimize outage probability is also provided. In [17], the authors propose a hop-by-hop relay selection strategy for multi-hop CWN, which provides a lower outage probability and a higher network throughput. Refs. [16,17] effectively improve the network SE to a certain extent, but they neglect the energy efficiency (EE) issue of the CRN.

To deal with the issue of energy inefficiency in CRN, simultaneous wireless information and power transfer (SWIPT) techniques are investigated in [18,19,20]. As mentioned in [21,22], SWIPT enables the simultaneous transmission of signals and energy through radio frequency signals. Thus, it can effectively extend the lifetime of energy-constrained wireless networks. Generally, there are two protocols with SWIPT techniques, namely, time switching and power splitting (PS) protocols. With the characteristics of SWIPT and CRN, introducing SWIPT into CRN, named SWIPT-enabled CRN, presents a promising solution for achieving energy-efficient and high-performance transmission, while improving the spectrum utilization [20].

Except for the SWIPT, optimal power allocation (OPA) is an effective method to improve the network SE and achieve energy-efficient transmission. For example, ref. [23] integrates the bidirectional relay, full-duplex, and SWIPT techniques, and investigates an alternating optimization algorithm to deal with the EE maximization problems, thus improving the network’s SE, maximizing the network’s EE, and prolonging the network’s lifetime. Ref. [24] proposes a multi-user time-power resource allocation algorithm, thus improving the network throughout. Ref. [25] uses SWIPT-PS protocol to investigate a joint resource allocation problem under power control, thus maximizing the minimum transmission rate for the SUs.

To provide an overview of the above studies, we can find that the BMR technique can reduce power consumption and improve the network SE. However, the traditional BMR selection algorithms usually fail to consider the SNR and global channel gain, simultaneously. The global channel gain means the channels between the relay node and the two receive nodes, rather than one of the receive nodes. At the same time, these relay selection algorithms mostly focus on using the relay link to achieve a bidirectional relay transmission (BRT). This means that they neglect the DL, which may be better to achieve a bidirectional direct transmission (BDT). Thus, they miss the chance to achieve an adaptive transmission with considering the relay link and DL, simultaneously. With the adaptive transmission, the network can switch between the BRT and BDT modes to achieve a higher transmission rate. Both of these two reasons will decrease the network SE with BMR transmission. In addition, with the combination of the BMR technique and CWN to construct a CRN, we can alleviate the issue of spectrum scarcity and further improve the network SE. But the existing studies usually focus more on improving the network SE, while relatively less attention is put on the energy-constrained relay nodes and the network EE. Furthermore, SWIPT is an effective method to solve the energy-constrained problem to extend the network lifetime and improve the network EE. What is more, the OPA also can effectively improve the network SE and EE.

Based on the above literature review and analyses, in this paper, in order to effectively improve the SE and EE of the wireless network, and also to tackle the insufficiency of spectrum resources and the issues of energy-constrained nodes with the consideration of the above reasons, we design an energy-efficient adaptive bidirectional transmission strategy in the SWIPT-enabled CRN. At the same time, different from the mentioned studies, our designed strategy considers the BMR, the DL, the CWN, the SWIPT, and the OPA simultaneously. To better elucidate the distinctions between our work and related studies, a detailed comparison is provided in Table 1. With Table 1, the main contributions of this paper can be further summarized as follows.

- To improve the network SE with BMR transmission, we propose to select an energy-constrained best relay node according to the received SNR of the relay node and the global channel gain from the relay node to SUs simultaneously to achieve a better BRT.
- To further improve the network SE with BMR transmission, we propose to consider both the relay link and DL and design a total transmit power threshold (TTPT) determining algorithm to find the TTPT, which lets the transmission rates of the BRT be equal to the BDT. Through the TTPT, the network can switch between the BRT and BDT modes and achieve an adaptive transmission to achieve a higher transmission rate.
- To address the energy constraints of the relay node and improve the network EE with the CRN, we propose to employ the energy-constrained best relay node to transmit signals by the SWIPT-PS protocol in the SWIPT-enabled CRN to extend the network lifetime.
- To further improve the network SE and EE with the CRN, we formulate an optimization problem under the constraint of the PUs interference threshold and obtain the analytical expressions of each node’s optimal power to maximize the transmission rate.

## 2. System Model

In this paper, the SWIPT-enabled CRN model as in [18,20] is considered. As in [18], all nodes are half-duplex and equipped with a single antenna. All nodes have perfect channel state information (CSI). All the nodes’ noise is modeled as addictive white Gaussian noise (AWGN) with zero mean and $\sigma^{2}$ variance. All signal and interference channels are independent of each other. And all channels undergo the quasi-statically symmetric complex Gaussian distribution. At the same time, as stated in [26], the SWIPT-enabled CRN can relieve the constraints of energy-constrained and spectrum resources for massive and small-sized nodes. Therefore, it can also be used in a variety of application scenarios, such as in the fields of IoT and UAV networks.

**Figure 1:**
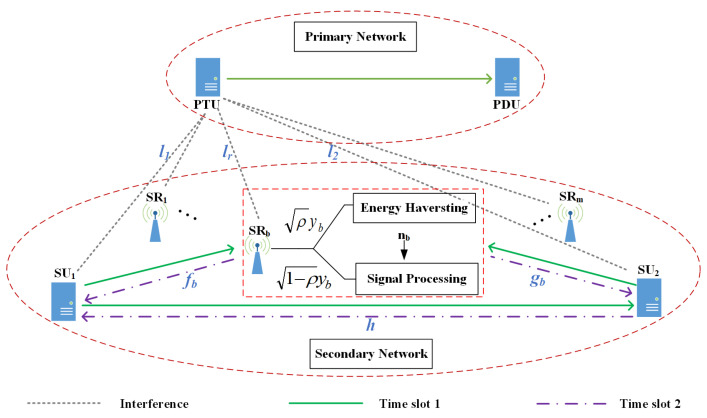
SWIPT-enabled CRN model.

With the above system setting, the SWIPT-enabled CRN model is shown in Figure 1. From Figure 1, we can find that it consists of a primary network and a secondary network. In the primary network, there is one primary transmit user (PTU) and one primary destination user (PDU). The PTU and PDU can transmit signals by direct transmission. In the secondary network, there are two SUs ($SU_{1}$, $SU_{2}$) and *m* energy-constrained relay nodes $SR_{k}$, where k=1,2,…,m. The $SR_{k}$ can transmit signals with the amplify-and-forward (AF) protocol [27]. The $SU_{1}$ and $SU_{2}$ can both act as transmit and receive nodes to exchange signals. And they can achieve bidirectional transmission through the energy-constrained best relay node $SR_{b}$ or the DL with the adaptive transmission. Namely, $SU_{1}$ and $SU_{2}$ can achieve bidirectional transmission through the BRT or the BDT modes. At the same time, the node which shows superiority in the consideration of both SNR and global channel gain will be $SR_{b}$. In addition, $SR_{b}$ will split the received signals into two parts with the SWIPT-PS protocol, namely, one part for energy harvesting (EH) and the other for signal processing (SP) [13].

**Figure 2:**
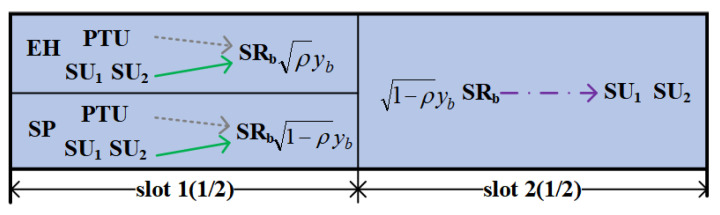
Time slots model.

To better show the transmission process of the SWIPT-enabled CRN model, the specific time slots model of the signal transmission is given in Figure 2. In Figure 1 and Figure 2, the green solid line and purple dashed line respectively represent the transmit signals in time slots 1 and 2. At the same time, the gray dashed line represents the interference signals. With Figure 1 and Figure 2, we can further assume that the signals sent by the PTU, $SU_{1}$ and $SU_{2}$ are respectively $x_{p}$, $x_{1}$, and $x_{2}$ with $E\left\{{\left|x_{p}\right|}^{2}\right\}=E\left\{{\left|x_{1}\right|}^{2}\right\}=E\left\{{\left|x_{2}\right|}^{2}\right\}=1$. The transmit powers of PTU, $SU_{1}$, and $SU_{2}$ are respectively $P_{P}$, $P_{1}$, and $P_{2}$. The channel coefficient from $SU_{1}$ to $SU_{2}$ is *h* with $h∼CN\left(0,\sigma_{h}^{2}\right)$. The channel coefficients from $SU_{1}$ and $SU_{2}$ to $SR_{b}$ are $f_{b}$ and $g_{b}$ with $f_{b}∼CN\left(0,\sigma_{1}^{2}\right)$ and $g_{b}∼CN\left(0,\sigma_{2}^{2}\right)$, respectively. The interference channel coefficients from PTU to $SU_{1}$, $SU_{2}$, and $SR_{b}$ are respectively $l_{1}$, $l_{2}$, and $l_{r}$ with $l_{1}∼CN\left(0,\sigma_{p_{1}}^{2}\right)$, $l_{2}∼CN\left(0,\sigma_{p_{2}}^{2}\right)$, and $l_{r}∼CN\left(0,\sigma_{p_{r}}^{2}\right)$.

## 3. Best Relay Selection Design and Transmission Rate Analysis

In this section, the design of the best relay selection and the analysis of transmission rate are given.

### 3.1. Best Relay Selection Design

To achieve a better BRT, we select the $SR_{b}$ for signal transmission by comparing each node’s SNR and global channel gain. The specific best relay selection process of the BMR transmission can be expressed as follows:

**Step 1.** Let the received SNR threshold at the $SR_{k}$ be $\gamma_{th}$, the received SNR from the $SU_{1}$ to $SR_{k}$ be $\gamma_{1k}$, and the received SNR from the $SU_{2}$ to $SR_{k}$ be $\gamma_{2k}$. Then, by comparing $\gamma_{1k}$ and $\gamma_{2k}$ with $\gamma_{th}$, the relay nodes that satisfy $\left\{\gamma_{1k},\gamma_{2k}\right\}>\gamma_{th}$ will be the effective relay nodes. And these effective relay nodes can form a candidate relay set $M(SR_{k})$.

**Step 2.** Let the channel gains from the $SU_{1}$ and $SU_{2}$ to $SR_{k}$ be $f_{k}^{2}$ and $g_{k}^{2}$, respectively. Then, the relay nodes that satisfy the following conditions will be the reliable relay nodes. And these reliable relay nodes can form a candidate relay set $N(SR_{k})$.

$$N\left(SR_{k}\right)=\left\{\begin{array}{c} A=\left(\max\left(f_{k}^{2}\right),\max\left(g_{k}^{2}\right)\right)\;or\\ B=\left(\max\left(f_{k}^{2}\right),g_{k}^{2}\;\mathrm{sub-maximum}\right)\;or\\ C=\left(\max\left(g_{k}^{2}\right),f_{k}^{2}\;\mathrm{sub-maximum}\right) \end{array}\right\}$$

**Step 3.** Let the relay node that is in both $M(SR_{k})$ and $N(SR_{k})$ be the best relay node. And these best relay nodes can form a best relay set $B\left(SR_{b}\right)$.

**Step 4.** With the $B\left(SR_{b}\right)$, it can further have the following situations:

(1) If $B\left(SR_{b}\right)=⌀$, return to step 1 to re-select;

(2) If $B\left(SR_{b}\right)\neq ⌀$ and there is only a single relay node in $B\left(SR_{b}\right)$, this node will be the best relay node $SR_{b}$;

(3) If $B\left(SR_{b}\right)\neq ⌀$ and there are multiple relay nodes in $B\left(SR_{b}\right)$, the node with the maximum SNR in step 1 will be the best relay node $SR_{b}$.

### 3.2. Transmission Rate Analysis

In this paper, we consider the bidirectional transmission situation to improve the network SE and time slot utilization, thus the analysis of bidirectional signal transmission is considered. In such case, the total transmission rate in this paper is defined as $R_{T}=R_{1}+R_{2}$, where $R_{1}$ and $R_{2}$ are the transmission rates in two directions [28]. Then, the total transmission rate of the BRT is $R_{T}^{s}=R_{1}^{s}+R_{2}^{s}$, and the total transmission rate of the BDT is $R_{T}^{d}=R_{1}^{d}+R_{2}^{d}$. With the $R_{T}^{s}$ and $R_{T}^{d}$, we can further define a function of $f(P_{T})$ with $f\left(P_{T}\right)=R_{T}^{s}\left(P_{T}\right)-R_{T}^{d}\left(P_{T}\right)$. The $P_{T}$ is the network total transmit power. Based on the function of $f(P_{T})$ and also to achieve the adaptive transmission, in this paper, when $f(P_{T})>0$, it means the total transmission rate of the BRT is higher, then we can let the network use the BRT to transmit the signal; otherwise, we can let the network use the BDT to transmit the signal. With the definition of function $f(P_{T})$, the specific analysis of transmission rate can be given in the following parts.

1. When $f(P_{T})>0$, from Figure 1 and Figure 2, we can know that in time slot 1, $SU_{1}$ and $SU_{2}$ respectively transmit signals to the $SR_{b}$. The signal received by the $SR_{b}$ can be expressed as

(1) $$y_{b}=\sqrt{P_{1}}f_{b}x_{1}+\sqrt{P_{2}}g_{b}x_{2}+\sqrt{P_{P}}l_{r}x_{p}+n_{r}$$

where $n_{r}$ is the noise at the $SR_{b}$ with $n_{r}∼CN\left(0,\sigma_{r}^{2}\right)$. With the SWIPT-PS protocol, the power splitting coefficient of the $SR_{b}$ is ρ with 0<ρ<1 [24]. Therefore, according to (1), the harvested energy by the $SR_{b}$ can be expressed as

(2) $$E_{R}=\frac{1}{2}\rho \eta \left(P_{1}{\left|f_{b}\right|}^{2}+P_{2}{\left|g_{b}\right|}^{2}+P_{P}{\left|l_{r}\right|}^{2}+\sigma_{r}^{2}\right)$$

where η is the energy conversion efficiency with 0<η<1 [24]. As in (2), to simplify the analysis, the linear energy harvesting model as in [23,24] is considered in this paper. Actually, the non-linear energy harvesting model can reflect the practical non-linear energy harvesting structures more accurately, and the non-linear one can be considered in our further work. According to [29], the whole harvested energy $E_{R}$ by the $SR_{b}$ can be used for signal transmission in time slot 2. Therefore, the transmit power at the $SR_{b}$ in time slot 2 can be expressed as

(3) $$P_{R}=\frac{E_{R}}{1/2}=\rho \eta \left(P_{1}{\left|f_{b}\right|}^{2}+P_{2}{\left|g_{b}\right|}^{2}+P_{P}{\left|l_{r}\right|}^{2}+\sigma_{r}^{2}\right)$$

From (3), we can find that with the SWPIT-PS protocol, the $SR_{b}$ does not increase the network total transmit power. In such a case, without the OPA, $P_{1}=P_{2}=\frac{1}{2}P_{T}$ can be obtained. Then, with the same network total transmit power, the transmission rate can be increased because more transmit powers can be allocated to $SU_{1}$ and $SU_{2}$ with (3).

In time slot 2, the $SR_{b}$ transmits the amplified signal to the $SU_{1}$ and $SU_{2}$ with AF protocol. In such case, the signals received by $SU_{1}$ and $SU_{2}$ can be expressed as

(4) $$\begin{array}{cc} y_{1}= & f_{b}\hat{y_{b}}+\sqrt{P_{P}}l_{1}x_{p}=\beta \sqrt{1-\rho }\sqrt{P_{1}}{f_{b}}^{2}x_{1}+\beta \sqrt{1-\rho }\sqrt{P_{2}}f_{b}g_{b}x_{2}\\ \; & +\beta \sqrt{1-\rho }\sqrt{P_{P}}f_{b}l_{r}x_{p}+\sqrt{P_{P}}l_{1}x_{p}+\beta \sqrt{1-\rho }f_{b}n_{r}+\beta f_{b}n_{b}+n_{1} \end{array}$$

(5) $$\begin{array}{cc} y_{2}= & g_{b}\hat{y_{b}}+\sqrt{P_{P}}h_{2}x_{p}=\beta \sqrt{1-\rho }\sqrt{P_{2}}g_{b}^{2}x_{2}+\beta \sqrt{1-\rho }\sqrt{P_{1}}f_{b}g_{b}x_{1}\\ \; & +\beta \sqrt{1-\rho }\sqrt{P_{P}}g_{b}l_{r}x_{p}+\sqrt{P_{P}}l_{2}x_{p}+\beta \sqrt{1-\rho }g_{b}n_{r}+\beta g_{b}n_{b}+n_{2} \end{array}$$

where $\hat{y_{b}}=\beta \left(\sqrt{1-\rho }y_{b}+n_{b}\right)$, $n_{1}$ and $n_{2}$ are respectively the noises at $SU_{1}$ and $SU_{2}$ with $n_{1}∼CN\left(0,\sigma_{1}^{2}\right)$ and $n_{2}∼CN\left(0,\sigma_{2}^{2}\right)$, and β is the amplification factor with $\beta =\sqrt{\frac{P_{R}}{\left(1-\rho \right)\left(P_{1}f_{b}^{2}+P_{2}g_{b}^{2}+P_{P}{\left|h_{r}\right|}^{2}+{\sigma_{r}}^{2}\right)}}$. According to (3), β can be further given by $\beta =\sqrt{\frac{\rho \eta }{1-\rho }}$ [30].

For the nodes with the perfect CSI, the self-signals of $SU_{1}$ and $SU_{2}$ can be cancelled [9]. After cancelling self-signals, the signals received at $SU_{1}$ and $SU_{2}$ can be rewritten as

(6) $$\hat{y_{1}}\;=\;\beta \sqrt{1-\rho }\sqrt{P_{2}}f_{b}g_{b}x_{2}\;+\;\beta \sqrt{1-\rho }\sqrt{P_{P}}f_{b}l_{r}x_{p}\;+\;\sqrt{P_{P}}l_{1}x_{p}\;+\;\beta \sqrt{1-\rho }f_{b}n_{r}\;+\;\beta f_{b}n_{b}\;+\;n_{1}$$

(7) $$\hat{y_{2}}\;=\;\beta \sqrt{1-\rho }\sqrt{P_{1}}f_{b}g_{b}x_{1}\;+\;\beta \sqrt{1-\rho }\sqrt{P_{P}}g_{b}l_{r}x_{p}\;+\;\sqrt{P_{P}}l_{2}x_{p}\;+\;\beta \sqrt{1-\rho }g_{b}n_{r}\;+\;\beta g_{b}n_{b}\;+\;n_{2}$$

Based on (6) and (7), the transmission rates of $SU_{1}$ and $SU_{2}$ can be expressed as

(8) $$R_{1}^{s}=\frac{1}{2}\log_{2}\left(1+\frac{\left(1-\rho \right)\rho \eta P_{2}{\left|f_{b}\right|}^{2}{\left|g_{b}\right|}^{2}}{\left(1-\rho \right)\rho \eta P_{P}{\left|f_{b}\right|}^{2}{\left|l_{r}\right|}^{2}+\left(1-\rho \right)P_{P}{\left|l_{1}\right|}^{2}+N_{1}}\right)$$

(9) $$R_{2}^{s}=\frac{1}{2}\log_{2}\left(1+\frac{\left(1-\rho \right)\rho \eta P_{1}{\left|f_{b}\right|}^{2}{\left|g_{b}\right|}^{2}}{\left(1-\rho \right)\rho \eta P_{P}{\left|g_{b}\right|}^{2}{\left|l_{r}\right|}^{2}+\left(1-\rho \right)P_{P}{\left|l_{2}\right|}^{2}+N_{2}}\right)$$

where $N_{1}=\left[\left(2-\rho \right)\rho \eta {\left|f_{b}\right|}^{2}+\left(1-\rho \right)\right]\sigma^{2}$ and $N_{2}=\left[\left(2-\rho \right)\rho \eta {\left|g_{b}\right|}^{2}+\left(1-\rho \right)\right]\sigma^{2}$. With (8) and (9), the $R_{T}^{s}$ can be obtained.

2. When $f(P_{T})\leq 0$, from Figure 1 and Figure 2, we can know that the signals received by $SU_{1}$ and $SU_{2}$ can be expressed by

(10) $$\hat{z_{i}}=\sqrt{P_{j}}hx_{j}+\sqrt{P_{P}}l_{i}x_{p}+n_{i},\left\{i,j\right\}=\left\{1,2\right\},i\neq j$$

Based on (10), the transmission rates of $SU_{1}$ and $SU_{2}$ can be expressed as

(11) $$R_{i}^{d}=\log_{2}\left(1+\frac{P_{j}h^{2}}{P_{P}l_{i}^{2}+\sigma_{i}^{2}}\right),\left\{i,j\right\}=\left\{1,2\right\},i\neq j$$

With (11), $R_{T}^{d}$ can be obtained.

## 4. Adaptive Transmission Strategy Design and Outage Probability Analysis

In this section, after the best relay selection, the design of the adaptive transmission strategy and the analysis of outage probability are given.

### 4.1. Adaptive Transmission Strategy Design

Based on the definition of the function $f(P_{T})$, we can know that the network can switch between the BRT and BDT modes. Under the equal power allocation, $P_{1}=P_{2}=\frac{1}{2}P_{T}$ can be obtained. In such a case, $f(P_{T})$ can be expressed as

(12) $$\begin{array}{cc} f(P_{T})= & \frac{1}{2}\log_{2}\left(1+\frac{\left(1-\rho \right)\rho \eta \frac{1}{2}P_{T}{\left|f_{b}\right|}^{2}{\left|g_{b}\right|}^{2}}{\left(1-\rho \right)\rho \eta P_{P}{\left|f_{b}\right|}^{2}{\left|l_{r}\right|}^{2}+\left(1-\rho \right)P_{P}{\left|l_{1}\right|}^{2}+N_{1}}\right)\\ \; & +\frac{1}{2}\log_{2}\left(1+\frac{\left(1-\rho \right)\rho \eta \frac{1}{2}P_{T}{\left|f_{b}\right|}^{2}{\left|g_{b}\right|}^{2}}{\left(1-\rho \right)\rho \eta P_{P}{\left|g_{b}\right|}^{2}{\left|l_{r}\right|}^{2}+\left(1-\rho \right)P_{P}{\left|l_{2}\right|}^{2}+N_{2}}\right)\\ \; & -\log_{2}\left(1+\frac{\frac{1}{2}P_{T}h^{2}}{P_{P}l_{i}^{2}+\sigma_{i}^{2}}\right)-\log_{2}\left(1+\frac{\frac{1}{2}P_{T}h^{2}}{P_{P}l_{i}^{2}+\sigma_{i}^{2}}\right) \end{array}$$

In order to achieve the adaptive transmission conveniently, with (12), we design a TTPT determining algorithm to find the TTPT $P_{th}$, which lets the $R_{T}^{s}$ be equal to the $R_{T}^{d}$, namely, $f\left(P_{th}\right)=R_{T}^{s}\left(P_{th}\right)-R_{T}^{d}\left(P_{th}\right)=0$. At the same time, with the dichotomy and the zero-point theorem, we can find the solution of $f(P_{th})=0$. Based on it, we can just compare the network total transmit power $P_{T}$ with $P_{th}$ to determine the network final transmission mode. Specifically, for $P_{T}\in \left(P_{T_{l}},P_{T_{u}}\right)$, if $f\left(P_{T_{l}}\right)f\left(P_{T_{u}}\right)<0$, then the threshold $P_{th}\in \left(P_{T_{l}},P_{T_{u}}\right)$ that makes $f(P_{th})=0$ can be found. In such a case, we set $P_{T_{m}}=\frac{P_{T_{u}}+P_{T_{l}}}{2}$ and calculate $f(P_{T_{m}})$. Then, if $f\left(P_{T_{l}}\right)f\left(P_{T_{m}}\right)<0$, a threshold $P_{th}\in \left(P_{T_{l}},P_{T_{m}}\right)$ that makes $f(P_{th})=0$ can be found. Otherwise, if $f\left(P_{T_{m}}\right)f\left(P_{T_{u}}\right)<0$, a threshold $P_{th}\in \left(P_{T_{m}},P_{T_{u}}\right)$ that makes $f(P_{th})=0$ can be found. Repeat the above algorithm until $P_{th}\in \left(P_{T_{l}},P_{T_{u}}\right)$, where $(P_{T_{l}},P_{T_{u}})<\tau$. From this method, we can find a TTPT $P_{th}$ that makes $R_{T}^{s}\left(P_{th}\right)=R_{T}^{d}\left(P_{th}\right)$. Based on the above analysis, the detailed algorithm can be seen as Algorithm 1.

According to Algorithm 1, $P_{th}$ can be found. With $P_{th}$, the adaptive bidirectional transmission strategy can be expressed as follows.

**Case 1.** When $P_{T}>P_{th}$, $R_{T}^{s}>R_{T}^{d}$ can be obtained, which means the total transmission rate of BRT is bigger than BDT, and the network still use BRT for signal transmission.

**Case 2.** When $P_{T}\leq P_{th}$, $R_{T}^{s}\leq R_{T}^{d}$ can be obtained, which means the total transmission rate of BDT is not smaller than BRT. Then, the relay node will change to dormant, and the network changes to using BDT for signal transmission.
**Algorithm 1:** TTPT determining algorithm.
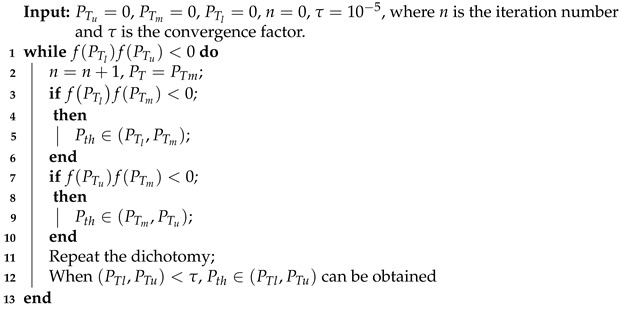


### 4.2. Outage Probability Analysis

According to [31], when $\left\{\min\left(\gamma_{1},\gamma_{2}\right)<\gamma_{th}\right\}$, the network will be outage. $\gamma_{1}$ and $\gamma_{2}$ are the SNRs at $SU_{1}$ and $SU_{2}$, respectively, and $\gamma_{th}$ is the SNR threshold to avoid a network outage. Therefore, the network outage probability can be given as

(13) $$P_{\mathrm{out}}=P_{r}\left\{\min\left(\gamma_{1},\gamma_{2}\right)<\gamma_{\mathrm{th}}\right\}$$

Assuming $\gamma_{1}\leq \gamma_{2}$, with the found $P_{th}$, the analysis of the outage probability can be presented as follows.

1. When $P_{T}>P_{th}$, from (8), the SNR at $SU_{1}$ can be expressed as $\gamma_{1}^{s}=\frac{\left(1-\rho \right)\rho \eta P_{2}{\left|f_{b}\right|}^{2}{\left|g_{b}\right|}^{2}}{X+N_{1}}$, where $X=\left(1-\rho \right)\rho \eta P_{P}{\left|f_{b}\right|}^{2}{\left|l_{r}\right|}^{2}+\left(1-\rho \right)P_{P}{\left|l_{1}\right|}^{2}$. Based on (13) and $\gamma_{1}^{s}$, the outage probability at $SU_{1}$ can be expressed as

(14) $$\begin{array}{cc} P_{out_{1}}^{s} & =P_{r}\left\{\gamma_{1}^{s}<\gamma_{th}\right\}\\ & =P_{r}\left\{\frac{\left(1-\rho \right)\rho \eta P_{2}{\left|f_{b}\right|}^{2}{\left|g_{b}\right|}^{2}}{\left(1-\rho \right)\rho \eta P_{P}{\left|f_{b}\right|}^{2}{\left|l_{r}\right|}^{2}+\left(1-\rho \right)P_{P}{\left|l_{1}\right|}^{2}+N_{1}}<\gamma_{th}\right\}\\ & =P_{r}\left\{w<\frac{\gamma_{th}}{\left(1-\rho \right)\rho \eta P_{2}}\left[\left(1-\rho \right)P_{P}{\left|l_{1}\right|}^{2}+N_{1}\right]\right\} \end{array}$$

where $w={\left|f_{b}\right|}^{2}\left({\left|g_{b}\right|}^{2}-\frac{\gamma_{th}P_{P}{\left|l_{r}\right|}^{2}}{P_{2}}\right)$. Meanwhile, the probability density function of the random variable *w* can be given as

(15) $$p_{w}\left(w\right)=\left\{\begin{array}{c} \frac{1}{\sigma_{1}^{2}\left(\sigma_{2}^{2}+a\sigma_{r}^{2}\right)}\int_{0}^{\infty }\frac{1}{v}e^{-\frac{v}{\sigma_{1}^{2}}-\frac{w}{\sigma_{2}^{2}}}dv,w>0\\ \frac{1}{\sigma_{1}^{2}\left(\sigma_{2}^{2}+a\sigma_{r}^{2}\right)}\int_{0}^{\infty }\frac{1}{v}e^{-\frac{w}{av\sigma_{r}^{2}}-\frac{v}{\sigma_{1}^{2}}}dv,w\leq 0 \end{array}\right.$$

Let $u={\left|g_{b}\right|}^{2}-\frac{\gamma_{th}P_{P}{\left|l_{r}\right|}^{2}}{P_{2}}$, and when u≤0, we can obtain

(16) $$P_{r}\left\{\gamma_{1}^{s}<\gamma_{th},u\leq 0\right\}=\frac{a\sigma_{r}^{2}}{\sigma_{2}^{2}+a\sigma_{r}^{2}}$$

where $a=\frac{\gamma_{th}P_{P}}{P_{2}}$. When u>0, we can obtain

(17) $$\begin{array}{cc} P_{r}\left\{\gamma_{1}^{s}<\gamma_{th},u>0\right\}= & \mu_{\sigma }-\mu_{\sigma }\sqrt{\frac{4c}{\sigma_{1}^{2}\sigma_{2}^{2}}}K_{1}\left(\sqrt{\frac{4c}{\sigma_{1}^{2}\sigma_{2}^{2}}}\right)\\ \; & +\frac{\mu_{\sigma }}{\sigma_{1}^{2}}\int_{0}^{\infty }\frac{b}{v+b}e^{-\frac{c}{v\sigma_{2}^{2}}-\frac{v}{\sigma_{1}^{2}}}dv \end{array}$$

where $K_{1}$ is the Bessel function of the second kind with $K_{1}\left(z\right)=\frac{z}{4}\int_{0}^{\infty }\frac{e^{-t-\frac{z^{2}}{4t}}}{t^{2}}dt$, $\mu_{\sigma }=\frac{\sigma_{2}^{2}}{\sigma_{2}^{2}+a\sigma_{r}^{2}}$, $b=\frac{\gamma_{th}P_{P}\sigma_{p_{1}}^{2}}{\rho \eta P_{2}\sigma_{2}^{2}}$, and $c=\frac{\gamma_{th}N_{1}}{\left(1-\rho \right)\rho \eta P_{2}}$. The integral part of (17) can be calculated according to [29]. Then, combining (16) and (17), the outage probability at $SU_{1}$ can be obtained as

(18) $$\begin{array}{cc} P_{out}^{s}= & \mu_{\sigma }-\mu_{\sigma }\sqrt{\frac{4c}{\sigma_{1}^{2}\sigma_{2}^{2}}}K_{1}\left(\sqrt{\frac{4c}{\sigma_{1}^{2}\sigma_{2}^{2}}}\right)+\frac{a\sigma_{r}^{2}}{\sigma_{2}^{2}+a\sigma_{r}^{2}}\\ \; & +\frac{\mu_{\sigma }}{\sigma_{1}^{2}}\int_{0}^{\infty }\frac{b}{v+b}e^{-\frac{c}{v\sigma_{2}^{2}}-\frac{v}{\sigma_{1}^{2}}}dv \end{array}$$

2. When $P_{T}\leq P_{th}$, from (11), the SNR at $SU_{1}$ can be expressed as $\gamma_{1}^{d}=\frac{P_{2}h^{2}}{P_{P}l_{1}^{2}+\sigma_{1}^{2}}$. Then, based on (13) and $\gamma_{1}^{d}$, the outage probability at $SU_{1}$ can be obtained as

(19) $$\begin{array}{cc} P_{out_{1}}^{d} & =P_{r}\left\{\gamma_{1}^{d}<\gamma_{th}\right\}=P_{r}\left\{\frac{P_{2}h^{2}}{P_{P}l_{1}^{2}+\sigma_{1}^{2}}<\gamma_{th}\right\}\\ & =P_{r}\left\{h^{2}<\frac{\gamma_{th}\left(P_{P}l_{1}^{2}+\sigma_{1}^{2}\right)}{P_{2}}\right\}\\ & =1-\frac{\sigma_{h}^{2}}{d\sigma_{p_{1}}^{2}+\sigma_{h}^{2}}e^{-\frac{\gamma_{th}\sigma_{1}^{2}}{P_{2}\sigma_{h}^{2}}} \end{array}$$

where $d=\frac{\gamma_{th}P_{P}}{P_{2}}$. The analysis of outage probability when $\gamma_{1}>\gamma_{2}$ can be omitted due to the similarity.

## 5. Optimal Power Allocation Design and Energy Efficiency Analysis

In this section, after the best relay selection and the adaptive transmission, the design of the OPA and the analysis of energy efficiency are given.

### 5.1. Optimal Power Allocation Design

According to [28], the EE can be defined as the ratio of the network total transmission rate and total transmit power. And the EE can be maximized through maximizing total transmission rate with the same total transmit power. In addition, according to [32], when optimizing each node’s power, the $P_{P}$ is assumed to be constant. At the same time, the OPA should consider the requirement of the interference threshold of PUs without affecting the normal communication of the PUs. In such case, in order to further improve the network EE and achieve energy-efficient transmission, each node’s power in the secondary network will be optimized through maximizing the total transmission rate with the same total transmit power under the requirement of PUs’ interference threshold *Q*. With the found $P_{th}$, the OPA can be presented as follows.

1. When $P_{T}>P_{th}$, the interference from $SU_{1}$ and $SU_{2}$ to PUs should satisfy $P_{1}{\left|l_{1}\right|}^{2}+P_{2}{\left|l_{2}\right|}^{2}=Q$ to assure the PUs’ performance. At the same time, according to [18,25], the transmit power of $SR_{b}$ is constrained not only by the harvested energy but also by the interference threshold of PUs. Therefore, with (3), the optimized transmit power of $SR_{b}$ can be expressed as $P_{R}^{s}=\min\left\{P_{R},\frac{Q}{{\left|l_{r}\right|}^{2}}\right\}$. Subsequently, let $Q_{1}=\frac{Q}{{\left|l_{1}\right|}^{2}}$ and $Q_{2}=\frac{Q}{{\left|l_{2}\right|}^{2}}$, to maximize the EE through maximizing the total transmission rate with the same total transmit power and the requirement of *Q*, the optimal problem can be described as

(20a) $$\begin{array}{cc} \max_{\varepsilon }\;\; & R_{1}^{s}+R_{2}^{s} \end{array}$$

(20b) $$\begin{array}{cc} s.t.\;\;\; & P_{1}=\varepsilon Q_{1} \end{array}$$

(20c) $$\begin{array}{cc} \; & P_{2}=\left(1-\varepsilon \right)Q_{2} \end{array}$$

(20d) $$\begin{array}{cc} \; & 0\leq \left\{P_{1},P_{2}\right\}\leq P_{T}^{\max} \end{array}$$

where ε is the power distribution factor under the interference threshold, and $P_{T}^{max}$ is the maximum transmit power. With the ε, the $P_{1}$ and $P_{2}$ also satisfy $P_{1}{\left|l_{1}\right|}^{2}+P_{2}{\left|l_{2}\right|}^{2}=Q$. Observing the optimal problem of (20a), it can be found that through finding the optimal ε under the Q, the total transmission rate can be maximized. Let $U^{s}=\frac{\left(1-\rho \right)\rho \eta P_{2}{\left|f_{b}\right|}^{2}{\left|g_{b}\right|}^{2}}{e_{1}+N_{1}}$ and $Z^{s}=\frac{\left(1-\rho \right)\rho \eta P_{1}{\left|f_{b}\right|}^{2}{\left|g_{b}\right|}^{2}}{e_{2}+N_{2}}$, and the optimal problem can be re-described as

(21a) $$\begin{array}{cc} \max_{\varepsilon }\;\; & \frac{1}{2}\left[\log_{2}\left(1+U^{s}\right)+\log_{2}\left(1+Z^{s}\right)\right] \end{array}$$

(21b) $$\begin{array}{cc} s.t.\;\;\; & P_{1}=\varepsilon Q_{1} \end{array}$$

(21c) $$\begin{array}{cc} \; & P_{2}=\left(1-\varepsilon \right)Q_{2} \end{array}$$

(21d) $$\begin{array}{cc} \; & 0\leq \left\{P_{1},P_{2}\right\}\leq P_{T}^{\max} \end{array}$$

where $e_{1}\;=\;\left(1\;-\;\rho \right)\rho \eta P_{P}{\left|f_{b}\right|}^{2}{\left|l_{r}\right|}^{2}\;+\;\left(1\;-\;\rho \right)P_{P}{\left|l_{1}\right|}^{2}$ and $e_{2}\;=\;\left(1\;-\;\rho \right)\rho \eta P_{P}{\left|g_{b}\right|}^{2}{\left|l_{r}\right|}^{2}\;+\;\left(1\;-\;\rho \right)P_{P}{\left|l_{2}\right|}^{2}$. Substituting (21b) and (21c) into (21a), the maximum of the total transmission rate can be obtained with the following equation:

(22) $$\max_{\varepsilon }\;\;\left[\log_{2}\left(1+U^{s}\right)+\log_{2}\left(1+Z^{s}\right)\right]=\frac{\partial \left[\log_{2}\left(1+U^{s}\right)+\log_{2}\left(1+Z^{s}\right)\right]}{\partial \varepsilon }=0$$

Solving (22), the optimal $\varepsilon =\frac{Q_{1}a_{2}-Q_{2}a_{1}+Q_{1}Q_{2}a_{1}a_{2}}{2Q_{1}Q_{2}a_{1}a_{2}}$ and $1-\varepsilon =\frac{Q_{2}a_{1}-Q_{1}a_{2}+Q_{1}Q_{2}a_{1}a_{2}}{2Q_{1}Q_{2}mn}$ can be obtained, where $a_{1}=\frac{\left(1-\rho \right)\rho \eta {\left|f_{b}\right|}^{2}{\left|g_{b}\right|}^{2}}{e_{1}+N_{1}}$ and $a_{2}=\frac{\left(1-\rho \right)\rho \eta {\left|f_{b}\right|}^{2}{\left|g_{b}\right|}^{2}}{e_{2}+N_{2}}$. Then, with (21b) and (21c), the optimal transmit powers can be finally obtained as

(23) $$\left\{\begin{array}{c} P_{1}^{s}=\frac{Q_{1}a_{1}-Q_{2}a_{2}+Q_{1}Q_{2}a_{1}a_{2}}{2Q_{2}a_{1}a_{2}}\\ P_{2}^{s}=\frac{Q_{2}a_{2}-Q_{1}a_{1}+Q_{1}Q_{2}a_{1}a_{2}}{2Q_{1}a_{1}a_{2}} \end{array}\right.$$

2. When $P_{T}\leq P_{th}$, $P_{1}{\left|l_{1}\right|}^{2}+P_{2}{\left|l_{2}\right|}^{2}=Q$ also should be satisfied. Also, let $Q_{1}=\frac{Q}{{\left|l_{1}\right|}^{2}}$ and $Q_{2}=\frac{Q}{{\left|l_{2}\right|}^{2}}$, to maximize the EE through maximizing the total transmission rate with the same total transmit power and the requirement of *Q*, and the optimal problem can be described as

(24a) $$\begin{array}{cc} \max_{\delta }\;\; & R_{1}^{d}+R_{2}^{d} \end{array}$$

(24b) $$\begin{array}{cc} s.t.\;\;\; & P_{1}=\delta Q_{1} \end{array}$$

(24c) $$\begin{array}{cc} \; & P_{2}=\left(1-\delta \right)Q_{2} \end{array}$$

(24d) $$\begin{array}{cc} \; & 0\leq \left\{P_{1},P_{2}\right\}\leq P_{T}^{\max} \end{array}$$

where δ is the power distribution factor under the interference threshold. With δ, $P_{1}$ and $P_{2}$ also satisfy $P_{1}{\left|l_{1}\right|}^{2}+P_{2}{\left|l_{2}\right|}^{2}=Q$. At the same time, it can be found that through finding the optimal δ under the Q, the total transmission rate can be maximized. Let $U^{d}=\frac{P_{2}h^{2}}{P_{P}l_{1}^{2}+\sigma_{1}^{2}}$ and $Z^{d}=\frac{P_{1}h^{2}}{P_{P}l_{2}^{2}+\sigma_{2}^{2}}$, and the optimal problem can be re-described as

(25a) $$\begin{array}{cc} \max_{\delta }\;\; & \log_{2}\left(1+U^{d}\right)+\log_{2}\left(1+Z^{d}\right) \end{array}$$

(25b) $$\begin{array}{cc} s.t.\;\;\; & P_{1}=\delta Q_{1} \end{array}$$

(25c) $$\begin{array}{cc} \; & P_{2}=\left(1-\delta \right)Q_{2} \end{array}$$

(25d) $$\begin{array}{cc} \; & 0\leq \left\{P_{1},P_{2}\right\}\leq P_{T}^{\max} \end{array}$$

Substituting (25b) and (25c) into (25a), the maximum of the total transmission rate can also be obtained with the following equation:

(26) $$\max_{\delta }\;\;\left[\log_{2}\left(1+U^{d}\right)+\log_{2}\left(1+Z^{d}\right)\right]=\frac{\partial \left[\log_{2}\left(1+U^{d}\right)+\log_{2}\left(1+Z^{d}\right)\right]}{\partial \delta }=0$$

Solving (26), the optimal $\delta =\frac{Q_{1}n-Q_{2}m+Q_{1}Q_{2}mn}{2Q_{1}Q_{2}mn}$ and $1-\delta =\frac{Q_{2}m-Q_{1}n+Q_{1}Q_{2}mn}{2Q_{1}Q_{2}mn}$ can be obtained, where $m=\frac{h^{2}}{P_{P}l_{1}^{2}+\sigma_{1}^{2}}$ and $n=\frac{h^{2}}{P_{P}l_{2}^{2}+\sigma_{2}^{2}}$. Then, with (25b) and (25c), the optimal transmit powers can be finally obtained as

(27) $$\left\{\begin{array}{c} P_{1}^{d}=\frac{Q_{1}n-Q_{2}m+Q_{1}Q_{2}mn}{2Q_{2}mn}\\ P_{2}^{d}=\frac{Q_{2}m-Q_{1}n+Q_{1}Q_{2}mn}{2Q_{1}mn} \end{array}\right.$$

### 5.2. Energy Efficiency Analysis

According to [28], the EE can be given as follows:

(28) $$\varsigma =\frac{R_{T}}{P_{T}}$$

Then, with the found $P_{th}$, the analysis of EE after OPA can be presented as follows.

1. When $P_{T}>P_{th}$, the total transmit power can be expressed as $P_{T}^{s}=P_{1}^{s}+P_{2}^{s}$. Combining (8), (9), (23) and (28), and $P_{T}^{s}$, the EE can be expressed as

(29) $$\varsigma^{s}=\frac{R_{T}^{s*}}{P_{T}^{s}}=\frac{R_{1}^{s*}+R_{2}^{s*}}{\frac{Q_{1}a_{2}-Q_{2}a_{1}+2Q_{1}Q_{2}a_{1}a_{2}}{2Q_{2}a_{1}a_{2}}+\frac{Q_{2}a_{1}-Q_{1}a_{2}+2Q_{1}Q_{2}a_{1}a_{2}}{2Q_{1}a_{1}a_{2}}}$$

where $\left\{\begin{array}{c} R_{1}^{s*}=\frac{1}{2}\log_{2}\left(1+\frac{\left(1-\rho \right)\rho \eta \left(Q_{1}a_{2}-Q_{2}a_{1}+2Q_{1}Q_{2}a_{1}a_{2}\right){\left|f_{b}\right|}^{2}{\left|g_{b}\right|}^{2}}{\left[\left(1-\rho \right)\rho \eta P_{P}{\left|f_{b}\right|}^{2}{\left|l_{r}\right|}^{2}+\left(1-\rho \right)P_{P}{\left|l_{1}\right|}^{2}+N_{1}\right]2Q_{2}a_{1}a_{2}}\right)\\ R_{2}^{s*}=\frac{1}{2}\log_{2}\left(1+\frac{\left(1-\rho \right)\rho \eta \left(Q_{2}a_{1}-Q_{1}a_{2}+2Q_{1}Q_{2}a_{1}a_{2}\right){\left|f_{b}\right|}^{2}{\left|g_{b}\right|}^{2}}{\left[\left(1-\rho \right)\rho \eta P_{P}{\left|g_{b}\right|}^{2}{\left|l_{r}\right|}^{2}+\left(1-\rho \right)P_{P}{\left|l_{2}\right|}^{2}+N_{2}\right]2Q_{1}a_{1}a_{2}}\right) \end{array}\right.$.

2. When $P_{T}\leq P_{th}$, the total transmit power can be expressed as $P_{T}^{d}=P_{1}^{d}+P_{2}^{d}$. Combining (11), (27) and (28), and $P_{T}^{d}$, the EE can be expressed as

(30) $$\varsigma^{d}=\frac{R_{T}^{d*}}{P_{T}^{d}}=\frac{R_{1}^{d*}+R_{2}^{d*}}{\frac{Q_{1}n-Q_{2}m+Q_{1}Q_{2}mn}{2Q_{2}mn}+\frac{Q_{2}m-Q_{1}n+Q_{1}Q_{2}mn}{2Q_{1}mn}}$$

where $R_{1}^{d*}=\log_{2}\left(1+\frac{\left(Q_{2}m-Q_{1}n+Q_{1}Q_{2}mn\right)h^{2}}{\left(P_{P}l_{1}^{2}+\sigma^{2}\right)2Q_{1}mn}\right)$ and $R_{2}^{d*}=\log_{2}\left(1+\frac{\left(Q_{1}n-Q_{2}m+Q_{1}Q_{2}mn\right)h^{2}}{\left(P_{P}l_{2}^{2}+\sigma^{2}\right)2Q_{2}mn}\right)$.

With the aforementioned analyses and designs based on the SWIPT-enabled CRN model, the implementation method of the proposed solution can be given as follows.

Firstly, we select the energy-constrained best relay node as the best relay selection process of the BMR transmission in Section 3.1 to achieve a better BRT transmission.

Secondly, based on the better BRT transmission, we use the TTPT determining algorithm to find the TTPT. Based on the TTPT, we can achieve the adaptive transmission as the adaptive transmission strategy in Section 4.1.

Thirdly, based on the two cases of the adaptive transmission strategy, according to the OPA method in Section 5.1, we optimize each node’s power under the constraint of the Pus interference threshold. Then, we let the nodes transmit signals with the optimized power.

At the same time, it should be noted that the implementation conditions of the physical devices are out of our research scope, and they can be checked in other related literature.

## 6. Numerical Results

In this section, simulation results of transmission rate, outage probability, and EE are presented to verify the effectiveness of the designed transmission strategy with Matlab 2022a. With the Matlab, the SWIPT-enabled CRN model and the signal transmission process and communication behavior can be highly restored [18,20]. And all the results are given with Monte Carlo simulation with 500 times loops in this paper. Specifically, the transmission performances of the following six transmission strategies, i.e., the designed energy-efficient adaptive bidirectional transmission strategy (namely, EEAB-SWIPT in the simulation), the designed energy-efficient adaptive bidirectional without SWIPT transmission strategy (namely, EEAB in the simulation), the designed adaptive bidirectional without SWIPT and OPA transmission strategy (namely, AB in the simulation), the two-way AF relay transmission strategy proposed in [30] (namely, TWBAF in the simulation), the best relay selection transmission strategy in [12] (namely, BRS in the simulation), and the non-collaborative transmission strategy (namely, NC in the simulation) are compared.

For the simulation, all channels are modeled as following a complex Gaussian distribution, and all node noises are assumed to be AWGN with a variance of $\sigma^{2}=1$. At the same time, all transmit signals have unit energy, i.e., $E\left\{{\left|x_{p}\right|}^{2}\right\}=E\left\{{\left|x_{1}\right|}^{2}\right\}=E\left\{{\left|x_{2}\right|}^{2}\right\}=1$. And the maximum transmit power is set to $P_{T}^{\max}=60$ W. What is more, in this paper, as in [25], the distances from PTU to the $SU_{1}$ and $SU_{2}$ are 5 m, while the distance between $SU_{1}$ and $SU_{2}$ is 10 m. At the same time, the multi-relay nodes are randomly distributed in the secondary network. The other simulation parameters are provided in Table 2.

### 6.1. Transmission Rate

**Figure 3:**
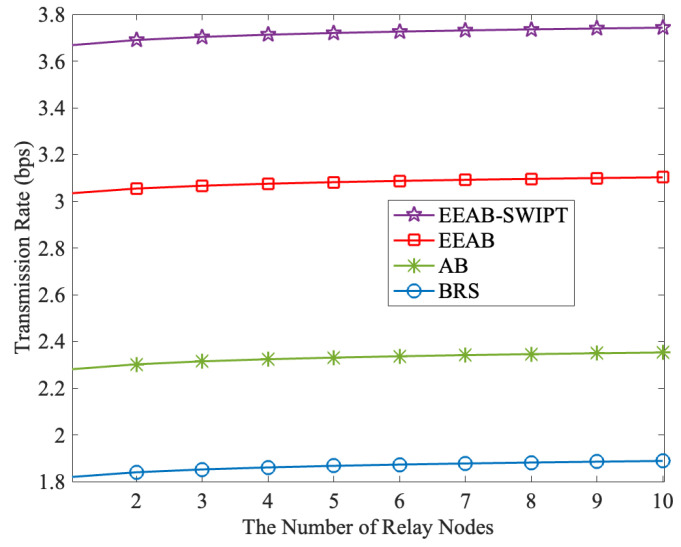
The transmission rate vs. the number of relay nodes.

Figure 3 shows the transmission rate with different numbers of relay nodes, where Q=15 dB and $P_{T}=30$ W. From it, we can find that the transmission rate increases as the number of relay nodes increases. When the number of relay nodes increases, a better $SR_{b}$ can be selected, thus increasing the transmission rate. Furthermore, when compared with the BRS, the transmission rates under EEAB-SWIPT, the EEAB, and the AB are enhanced, and EEAB-SWIPT obtains the highest transmission rate. For example, when the relay node’s number is 5, the transmission rates under BRS, AB, EEAB, and EEAB-SWIPT are 2.27 bps, 2.65 bps, 2.96 bps, and 3.72 bps, respectively. This is because the EEAB-SWIPT combines the SWIPT technique, the adaptive transmission, and the OPA, thus realizing the highest transmission rates. BRS only makes the best relay node selection with consideration of the part channel gain, and it also does not consider the adaptive transmission; thus, it has the lowest transmission rate.

**Figure 4:**
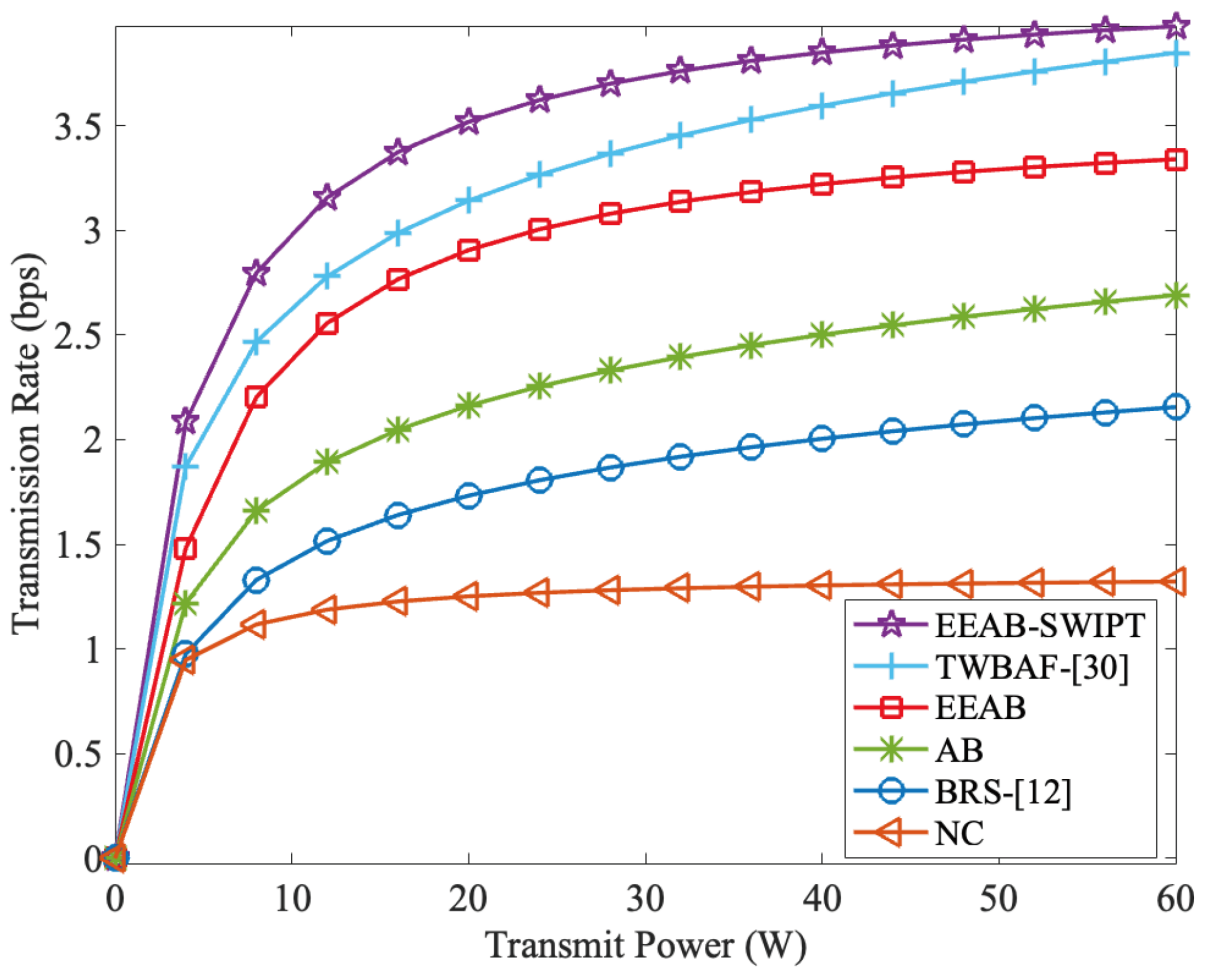
The transmission rate vs. the total transmit power.

Figure 4 shows the transmission rate with different total transmit powers, where Q=15 dB and the number of relay nodes is 5. From it, we can find that the transmission rate increases with the total transmit power increases. But when the total transmit power increases to a certain level, the increases in transmission rate become slow, which means the influence of the total transmit power becomes weak. Meanwhile, from Figure 4, the transmission rate of the EEAB-SWIPT is higher than that of any other transmission strategies. This is because our EEAB-SWIPT realizes adaptive transmission between the BRT and BDT based on the total transmit power to obtain a higher total transmission rate, which corresponds to the definition of function $f(P_{T})$. At the same time, our EEAB-SWIPT introduces the energy-constrained relay node to harvest energy so that a higher transmission rate can be obtained, which corresponds to Equation (3). In addition, our EEAB-SWIPT optimizes each node’s power, thus increasing the transmission rate with the same total transmit power. With Figure 3 and Figure 4, it can be found that the designed EEAB-SWIPT transmission strategy can effectively improve the network transmission rate.

**Figure 5:**
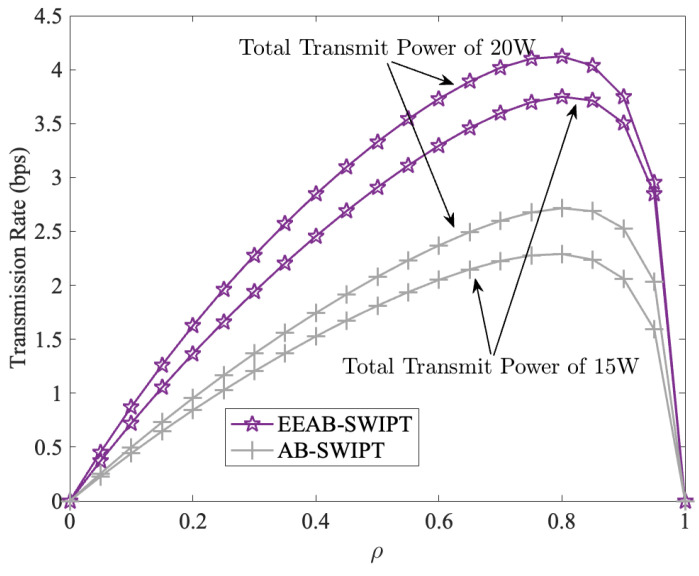
The transmission rate vs. the PS factor.

Figure 5 shows the transmission rate of EEAB-SWIPT and AB-SWIPT with a different PS coefficient ρ. The AB-SWIPT is our AB with SWIPT and without OPA. From it, we can find that no matter whether the total transmit power is 15 W or 20 W, the transmission rate first increases and then decreases. At the same time, the maximum transmission rate is achieved at around ρ=0.8. Because the energy harvested by the ${\mathrm{SR}}_{b}$ increases with ρ, while the SNR at the ${\mathrm{SR}}_{b}$ decreases with ρ, when ρ increases to a certain level, the transmission rate decreases with the increases in ρ. In addition, when compared with AB-SWIPT, EEAB-SWIPT obtains a higher transmission rate with the OPA. Furthermore, from Figure 5, for the same transmission strategy, the transmission rate increases with the total transmit power increases. This phenomenon corresponds to Figure 4.

### 6.2. Outage Probability

**Figure 6:**
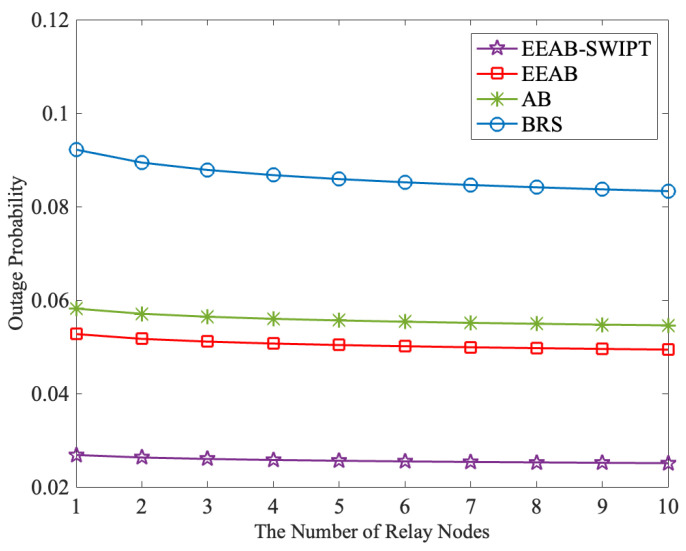
The outage probability vs. the number of relay nodes.

Figure 6 presents the outage probability with different numbers of relay nodes, where Q=15 dB and SNR = 22.5 dB. From it, we can find that the outage probability decreases slowly as the number of relay nodes increases. At the same time, corresponding to Figure 3, when the number of relay nodes increases to five, the influence of the relay node’s number on the outage probability becomes weak. At the same time, when compared with BRS, the AB achieves an adaptive transmission to obtain a lower outage probability. At the same time, the EEAB introduces the adaptive transmission and OPA to obtain a lower outage probability. Furthermore, with the adaptive transmission, SWIPT, and OPA, the outage probability can be further decreased, and the EEAB-SWIPT obtains the lowest outage probability. For example, when the relay node’s number is eight, the outage probabilities under BRS, AB, EEAB, and EEAB-SWIPT are 0.0.086, 0.055, 0.050, and 0.026, respectively.

**Figure 7:**
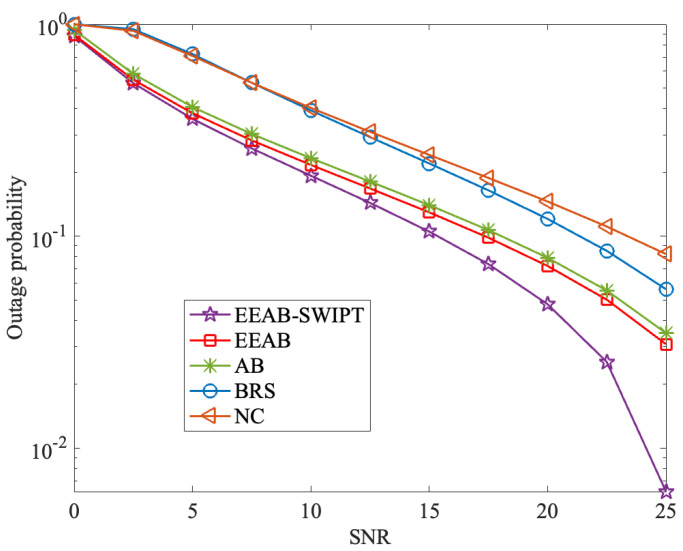
The outage probability vs. the SNR.

Figure 7 presents the outage probability with different SNRs, where Q=15 dB and the number of relay nodes is 6. From it, we can find that the outage probability decreases with the SNR increases. At the same time, the EEAB-SWIPT also obtains the lowest outage probability. For example, when SNR is 25 dB, the outage probabilities under NC, BRS, AB, EEAB, and EEAB-SWIPT are 0.082, 0.056, 0.035, 0.030, and 0.006, respectively. With Figure 6 and Figure 7, it can be found the designed EEAB-SWIPT transmission strategy effectively reduces the network outage probability.

**Figure 8:**
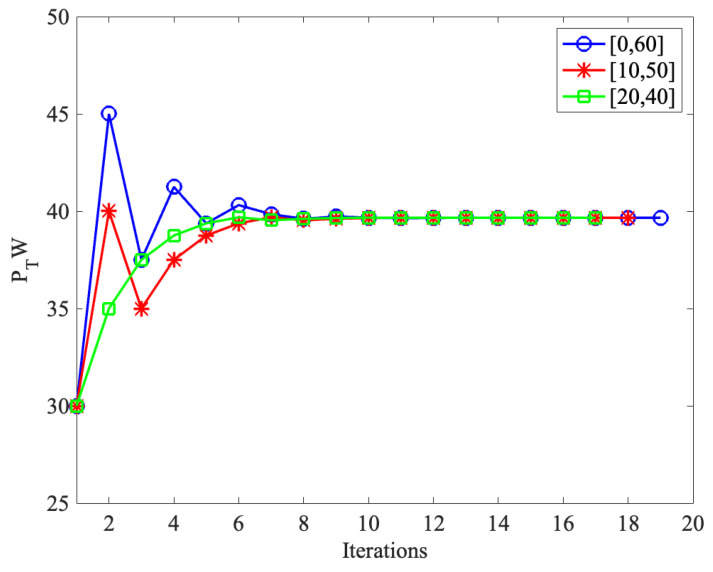
The convergence of the TTPT determining algorithm.

Figure 8 demonstrates the convergence of our proposed TTPT determining algorithm, where $P_{P}=10$ W, η=0.6, and ρ=0.5. In the figure, we select three different initial intervals, i.e., [0, 60], [10, 50], and [20, 40], to simulate its convergence behavior. From it, we can find that regardless of the initial interval, with almost nine iterations, the total transmit power $P_{T}$ stabilizes at around 39.7 W. This means that after nine iterations, the $P_{T}$, which lets the transmission rate of BRT be equal to that of BDT, can be found. It also shows the convergence of our proposed TTPT determining algorithm and the effectiveness of our proposed adaptive transmission strategy.

### 6.3. Energy Efficiency

**Figure 9:**
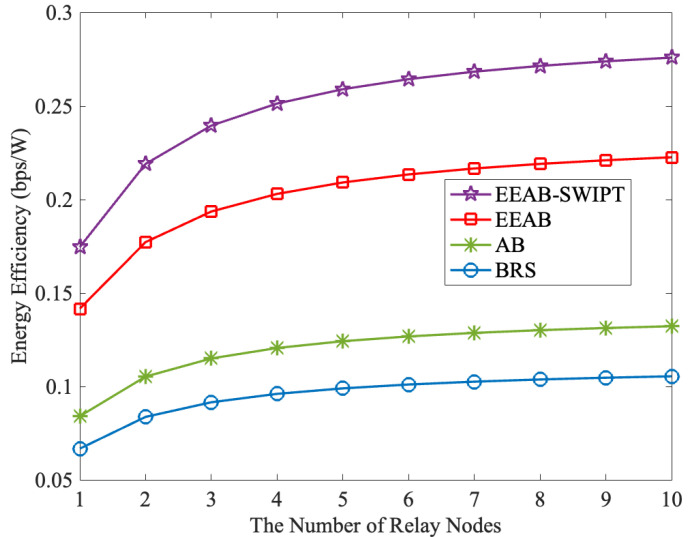
The EE vs. the number of relay nodes.

Figure 9 compares EE with a different number of relay nodes, where Q=12 dB and $P_{T}=12$ W. From it, we can find that the EE increases as the number of relay nodes increases. At the same time, corresponding to Figure 3, when the number of relay nodes increases to five, the influence of the relay node’s number gradually weakens. In addition, we can also find that our designed EEAB-SWIPT obtains the highest EE. This is for the same total transmit power; our designed EEAB-SWIPT has the highest transmission rate.

**Figure 10:**
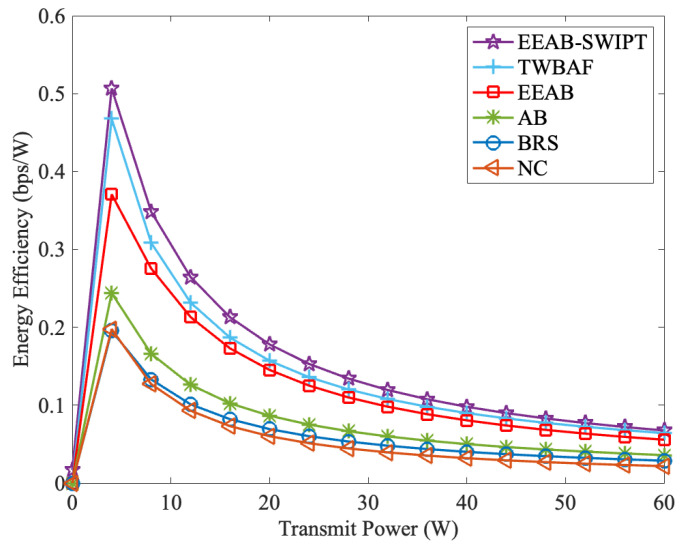
The EE vs. the total transmit power.

Figure 10 compares EE with different total transmit powers, where Q=12 dB and the number of relay nodes is six. From it, the following information can be obtained: (1) The EE first increases and then decreases with the increases in total transmit power. At the same time, the maximum EE is obtained at around 6 W. When the transmit power increases to a certain level, corresponding to Figure 4, the increases in transmit power cannot bring sufficient increases in transmission rate. That is, from (28), the growth in the denominator is higher than that of the numerator, and thus the network EE keeps decreasing. (2) Corresponding to Figure 4, with the increases in total transmit power, the EE under EEAB-SWIPT is the highest, and the NC is the lowest. This is because when compared with the other transmission strategies, our EEAB-SWIPT achieves the adaptive transmission, optimizes each node’s power allocation, and uses the SWIPT-PS protocol to transmit the energy-constrained node’s signal, simultaneously. With Figure 9 and Figure 10, we can find that the designed EEAB-SWIPT can effectively improve the network EE.

**Figure 11:**
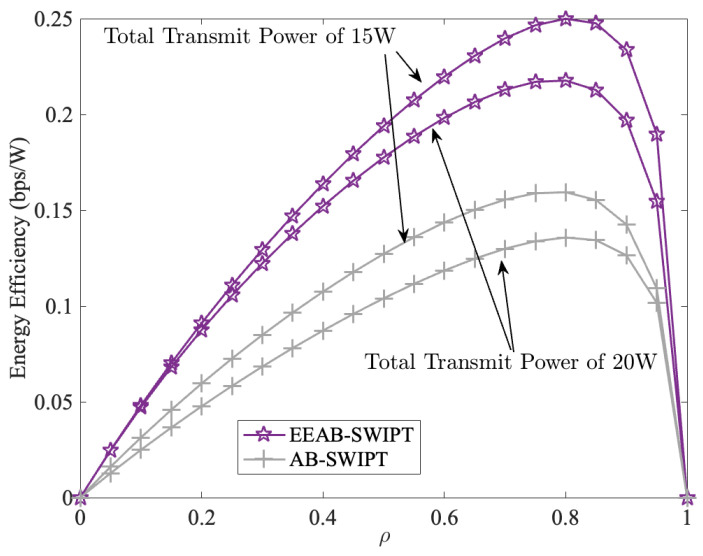
The EE vs. the PS factor.

Figure 11 compares EE of the EEAB-SWIPT and AB-SWIPT with a different PS coefficient ρ. From it, we can find that no matter whether the total transmit power is 15 W or 20 W, the EE first increases and then decreases. At the same time, the maximum EE is also achieved at around ρ=0.8, which corresponds to Figure 5. In addition, unlike in the Figure 5, the EE at 15 W is higher than that at 20 W under the same transmission strategy. This is because, as shown in Figure 10, after the EE reaches its maximum at 6 W, it then decreases with the total transmit power increases. Therefore, the EE at 20 W is lower than that at 15 W. However, when compared with the AB-SWIPT, the EEAB-SWIPT obtains a higher EE with the OPA, which can verify the effectiveness of our EEAB-SWIPT transmission strategy.

## 7. Conclusions

In this paper, we have designed an energy-efficient adaptive bidirectional transmission strategy in the SWIPT-enabled CRN. To design the transmission strategy, we first select an energy-constrained best relay node with the consideration of SNR and global channel gain. At the same time, we let the energy-constrained best relay node transmit a signal with the SWIPT-PS protocol. Then, with the selected energy-constrained best relay node, we further design a TTPT determining algorithm and design the adaptive bidirectional transmission strategy. With the designed transmission strategy, the network can achieve an adaptive transmission between the BRT and BDT modes to obtain a higher network SE. Furthermore, to further achieve energy-efficient transmission of the designed transmission strategy, we optimize the nodes’ power under the requirement of the PUs’ interference threshold and obtain the analytical expressions of the optimal power. Simulation results have shown that our designed transmission strategy can obtain higher SE and EE, and a lower outage probability, which verifies the effectiveness of our transmission strategy. In addition, considering the intelligent reflective surface can effectively improve the network’s EE and SE, it will be explored in our future work.

## Figures and Tables

**Table 1:** Comparisons with related studies.

Related References	BMR	DL	CWN	SWIPT	OPA
[6]		√			√
[8]	√				√
[11]	√		√		
[9,12]	√				
[14,16,17]	√		√		
[18,20]	√		√	√	
[19]		√		√	
[21]				√	
[22]		√		√	
[23]		√		√	√
[24,25]			√	√	√
This paper	√	√	√	√	√

**Table 2:** Simulation parameters.

Notation	Description	Values
$\sigma^{2}$	Noise power	1 W
SNR	Signal-to-noise ratio	[−10, 25] dB
Q	Interference threshold	(0, 15] dB
η	Energy conversion efficiency	0.6
ρ	PS factor	0.5
τ	Path loss factor	[2, 4]
$SR_{k}$	Relay number	[1, 10]
$P_{T}$	Total transmit power	[0, 60] W

## Data Availability

The data are unavailable due to privacy or ethical restrictions.

## Abbreviations

The following abbreviations are used in this manuscript:

CWN	Cognitive Wireless Network
CRN	Cognitive Relay Network
SE	Spectrum Efficiency
EE	Energy Efficiency
SWIPT	Simultaneous Wireless Information and Power Transfer
SNR	Signal-to-Noise Ratio
BMR	Bidirectional Multi-Relay
DL	Direct Link
BRT	Bidirectional Relay Transmission
BDT	Bidirectional Direct Transmission
TTPT	Total Transmit Power Threshold
OPA	Optimal Power Allocation
PU	Primary User
SU	Secondary User
PTU	Primary Transmit User
PDU	Primary Destination User
EH	Energy Harvesting
SP	Signal Processing
CSI	Channel State Information
PS	Power Splitting
AWGN	Addictive White Gaussian Noise
AF	Amplify and Forward
AB	Adaptive Bidirectional
EEAB	Energy-Efficient Adaptive Bidirectional
BRS	Best Relay Selection
NC	Non-Collaborative
